# Sustainability Evaluation of Polyhydroxyalkanoate Production from Slaughterhouse Residues Utilising Emergy Accounting

**DOI:** 10.3390/polym14010118

**Published:** 2021-12-29

**Authors:** Khurram Shahzad, Mohammad Rehan, Muhammad Imtiaz Rashid, Nadeem Ali, Ahmed Saleh Summan, Iqbal Muhammad Ibrahim Ismail

**Affiliations:** Centre of Excellence in Environmental Studies, King Abdulaziz University, Jeddah 21589, Saudi Arabia; dr.mohammad_rehan@yahoo.co.uk (M.R.); irmaliks@gmail.com (M.I.R.); nbahadar@kau.edu.sa (N.A.); asumman@kau.edu.sa (A.S.S.); iqbal30@hotmail.com (I.M.I.I.)

**Keywords:** biopolymers, sustainability, emergy accounting, polyhydoxyalkanoates, slaughterhouse residues

## Abstract

High raw material prices and rivalry from the food industry have hampered the adoption of renewable resource-based goods. It has necessitated the investigation of cost-cutting strategies such as locating low-cost raw material supplies and adopting cleaner manufacturing processes. Exploiting waste streams as substitute resources for the operations is one low-cost option. The present study evaluates the environmental burden of biopolymer (polyhydroxyalkanoate) production from slaughtering residues. The sustainability of the PHA production process will be assessed utilising the Emergy Accounting methodology. The effect of changing energy resources from business as usual (i.e., electricity mix from the grid and heat provision utilising natural gas) to different renewable energy resources is also evaluated. The emergy intensity for PHA production (seJ/g) shows a minor improvement ranging from 1.5% to 2% by changing only the electricity provision resources. This impact reaches up to 17% when electricity and heat provision resources are replaced with biomass resources. Similarly, the emergy intensity for PHA production using electricity EU27 mix, coal, hydropower, wind power, and biomass is about 5% to 7% lower than the emergy intensity of polyethylene high density (PE-HD). In comparison, its value is up to 21% lower for electricity and heat provision from biomass.

## 1. Introduction

Plastics are used in almost every aspect of daily life. These materials have opened new horizons for research and development in academia and the industry [1]. They are used for a variety of services such as food packaging [2], low-density supplies [3], resilient chemical commodities [4], specialised niche structures in electronic fabrication, as well as their use in the medical field as implants and scaffolds [5,6]. However, the collection of huge amounts of waste has resulted from indiscriminate usage and disposal. Because they are non-biodegradable, managing and disposing of them is becoming increasingly complex [7]. In 2018, the global plastic output hit 359 million tons, with 67.5 percent of plastics going unrecycled and contaminating ecosystems [8]. Moreover, plastic degrades pure habitats and damages humans and other organisms, necessitating a quick micromanagement. The World Health Organisation (WHO) has asked scientists to develop alternative ways and materials to replace traditional plastics to limit environmental and human exposures [9]. Biodegradable polymers, particularly polyhydroxy-alkanoates (PHAs) [10], have been proposed as a promising alternative to non-biodegradable polyethylene plastic polymers [11] and can help to reduce plastic overgrowth [12]. Aside from their useful properties and biodegradability, PHAs are one of the most promising materials of the 21st century. They are used for various applications in the agricultural sector, municipal engineering, pharmacology, and biomedicine [13].

Bioplastic production currently accounts for only 0.6 percent (2.05 million tons) of the total plastic output, but it is expected to increase to 2.44 million tons by 2023 [14]. The valorisation of various types of trash as a carbon source for a more cost-effective microbial PHA synthesis and environmental friendliness is the subject of exceptional research efforts worldwide [15]. However, several factors must be considered for commercial PHA production to be sustainable and beneficial. The economic cost, stable feedstock properties and composition, simplicity of collection, transport, storage, and, most importantly, no competition with food and feed streams are among these aspects [16].

The natural capital storage is being depleted because of direct and indirect resource demand (e.g., oil, chemicals, minerals, and the treatment of human waste) [17]. In this light, material circularity is a significant issue in the quest for fossil-based raw materials and energy alternatives. As an analytical and quantitative technique, eco-efficiency is used to examine sustainable development [18].

The food, drug, cosmetics, and leather sectors, among others, generate a massive amount of organic waste, which can be a source of harmful diseases [19]. Almost half of the livestock is turned into byproducts, which retain a meaningful amount of energy (tallow: 3.98E 04 J/g average, meat, and bone meal: 1.85E 04 J/g average) [20]. From a bio-refinery perspective, a wide range of products and commodities may be obtained by adequately managing animal slaughtering residues and byproducts (i.e., biochemicals and pharmaceutical products from the blood and gelatinous material, clothes from skins, etc.) through various processes [21,22]. A conventional refinery produces a variety of petrol-based fuels and products. In contrast, a bio-refinery uses residual biomass from agriculture, forests, or industries as a resource to produce fuels, power, heat, and value-added chemicals. Implementing a bio-refinery plan should utilise growing and dynamic existing systems rather than building brand new complexes [23].

Waste management systems are incredibly complicated, necessitating extra caution when making assumptions and methodological decisions. Because garbage is on the verge of consumption and creation (via recycling), the outcomes of this study are very much reliant on the assessment approach chosen. Furthermore, emergy accounting (EMA) may be viewed as a new and more complete measure of eco-efficiency. It calculates the environmental system effort required to support a product or service delivered by a specific process [20]. This methodology has never been reported for the ecological evaluation of PHA production as per the authors’ best knowledge. The effect of replacing energy provision resources from the business-as-usual scenario, i.e., the electricity mix from the grid and heat provision utilising natural gas, to various renewable energy ones will also be evaluated.

## 2. Methodology

As a methodological framework, EMA was employed in this study. Emergy is the energy (of a certain type) utilised in a system for transformations [24]. The limits in EMA are set at the level of the biosphere. The whole process demand and supply, including resource production, processing, and throwing away and the environmental inputs for the creation of storage and natural income flows (both renewable and non-renewable), which support the specified system directly or indirectly, are considered [25].

Ulgiati and Brown [26] indicate that the emergy input to a process is the result of all the resources and energy input traced back into all the processes or the chain of processes used in the process and expressed in the form of solar energy. Emergy is the measure of value for both energy and material resources at a standard basis, equivalent to the biosphere processes required to produce something [27]. The environmental services that are usually free of cost and outside the monetary economy and benefits of humans in the form of labour for processing the resource are embedded in the emergy value of the resource [28]. The emergy definition by Odum [29] is: “Emergy is the available energy (exergy) of one kind that is used up in transformations directly and indirectly to make a product or service”.

The value is a unit of solar equivalent energy articulated in solar emjoules, abbreviated as (seJ). The solar emjoule (sej) is the emergy unit, which refers to the consumption of available energy in the transformations to deliver a product or service or functioning of a process. Solar energy is undoubtedly the most abundant form of energy accessible for Earth’s activities. Thus, it is appropriate to utilise it as the reference kind of energy [30]. Resource production considers both evolutionary “trial and error” patterns and the diverse excellence of input flows, including solar, gravitational, and geothermal, each one of which is measured regarding its equivalency to solar radiation flow. Resource generation encapsulates the accessible energy flows invested inside the biosphere processes. The gradual development and the diverse qualities of input flows contributed by solar, geothermal, and gravitational, each computed concerning its equivalence to the solar radiation flow. Instead of merely “joule,” the word “em-joule” is noticeably more related to biosphere dynamics than the energy content indicated by a simple joule. As a result, the total emergy (*U*) operating a process includes the overall “ecological production cost” of goods or services, calculated by Equation (1) by adding all inputs going into the process [31].

(1) $$U=\sum_{i}fi\times tr_{i}\;\;\;\;\;\;\;\;\;\;\;\;\;\;\;\;\;i=1,2,\ldots .,\;n$$

where *U* stands for total energy, *fi* for the *i*-th input of energy or matter, UEV for the *i*-th inflow’s Unit Emergy Value, and *n* for the number of supporting inflows. The emergy investment per unit output may also be computed by using *U* and the process yield (*Y*), i.e., Unit Emergy Value, UEV, typically represented as sej/J or sej/g, as shown in Equation (2).

*UEV* = *U*/*Y* (2)

where *Y* is termed process yield, which is the output as energy (joules), mass (grams), or other suitable service units. When *Y* is expressed in joules, as is the case for all the energy flows, the *UEV* is referred to as “transformity” described as sej/J. The solar radiation’s transformity is set to 1 sej/J. All emergy values are calculated using a Global Emergy Baseline (GEB) as a reference, which is the total emergy accessible yearly to all biosphere activities. All UEVs in this work are connected to Brown et al., (2016)’s GEB [31], 12.0 E+24 seJ/yr. For emergy calculations, renewable inputs (solar radiations, wind, rain potential, geothermal) and non-renewable inputs (soil erosion) for a process, system, or region are calculated by utilising the area within the boundary conditions of the system under study. The boundary conditions of the system can be selected at the plant area, regional area, national level, and global level. The renewable as well as local non-renewable (soil erosion) depend on the spatial location of the system. Normally, system boundaries are set at the national level considering the national area for renewable and local non-renewable flow calculations [32].

EMA is a supply-side approach, since it considers direct and indirect contributions to systems and labour and service contributions. Indeed, evaluating an investment entails calculating the inescapable cost of resource replacement [32]. Since its inception, the concept of EMA has been widely applied in a variety of fields [33], including agriculture [34,35]), waste management [20], petroleum production [36], and coal mining [37].

The conversion of animal slaughtering waste or animal residues involves several sub-processes. These sub-processes are the hydrolysis, rendering, biodiesel production, and fermentation process. The detailed description of the process design [38], development [39], as well as economic [40] and ecological evaluation using SPI methodology has been published earlier [41,42]. The material and energy flows for the current study have been considered from the published literature [16]. The renewable inputs (sun, wind, and rain potential) are calculated and used for Austria. For emergy accounting, material and energy flows for a production capacity of 10,000 t/yr PHA production have been considered, and the following assumptions has been made:

- Animal slaughtering residues are considered as waste having zero emergy content. Although it does have emergy content, it can only be available when animal production (farming) is produced to produce meat. Therefore, this study assumes that all emergy content is assigned to the main product “meat”.
- Only mass and energy flows have been considered for analysis while infrastructure is out of the system boundary.
- Out of the renewable inputs (sun, wind, and rainfall), only the significant input has been accounted for to avoid double counting, considering that solar radiations are the sole energy source for wind movement and rainfall.
- Water input is supposed to be renewable, considering it is provided from the rain collected in a water reservoir or a lake or river.
- Water input has been converted to energy units by multiplying by the Gibbs free energy content of water.
- For wastewater treatment, only electricity consumption has been considered in the evaluation.
- The electrical demand is fulfilled using the European electricity mix (EU_27), while natural gas has been considered a source of heat provision.
- For wastewater treatment, electricity consumption has been considered as an input to the system.

**Figure 1:**
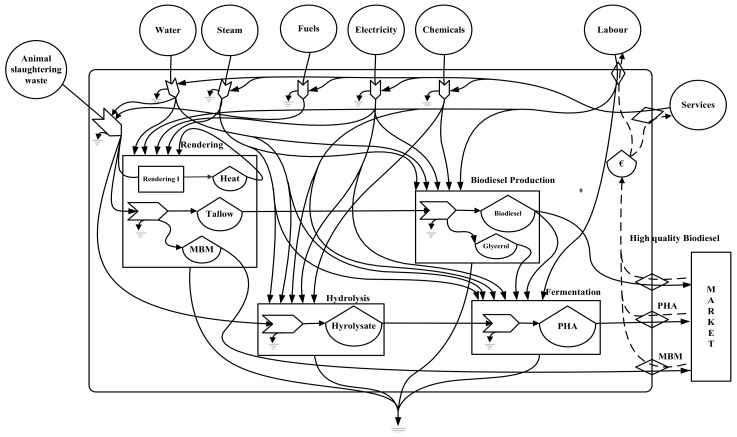
Emergy system diagram of PHA production process utilising slaughtering waste as starting material (the emergy symbol nomenclature is given Appendix B).

The assumptions for emergy accounting have been that renewable energy flows and water are used as a renewable input. Therefore, a system diagram is required to organise the evaluation and adequately account for inputs and outflows from the processes or systems. Figure 1 is the system diagram of the PHA production process utilising slaughtering waste as starting material input. It helps to construct evaluation tables of the actual flow of material, energy, labour, and services. All information (energy, material, labour, and environmental services) shown in the diagram are evaluated in their standard units (J, kg, m^3^, $, etc.). The information available in the form of evaluation table data is further processed to calculate different indices for PHA production.

Specific emergy (seJ/unit) for the material flows, and transformity (seJ/J) for the energy flows is the amount of emergy necessary to create one unit of each input. It is a quality factor measuring the ecological assistance intensity supplied by the planet during the development of each input [43]. The emergy intensity values for different input flows have been shown in Table 1. The reference sources for the unit emergy intensity values have been written as a footnote at the end of the table.

## 3. Results and Discussion

The results of the emergy accounting analysis have been shown in the emergy evaluation table for each sub-process, one by one, as follows:

### 3.1. Transportation Phase

The emergy content calculated for the transportation phase is 1.78E+19 seJ/yr and 1.19E+19 seJ/yr, with (L&S) and without labour and services (L&S), respectively. The emergy accounting for the transportation phase has been shown in Table 2.

### 3.2. Rendering I

It is assumed that the “rendering facility processes 100,000 t/yr slaughtering residue, and it operates 250 day/yr”. This facility requires 3.28E+02 h/yr to process 4.87E+03 t/yr of condemned material in the rendering I facility. This time is equivalent to the labour force of 2.05E-01person/yr. Therefore, the sum of share of the transportation service share and utility cost constitutes the service input for rendering I, which is 2.44E+05 €/yr. The inventory input and emergy content calculated for rendering I is given in Table 3.

**Figure 2:**
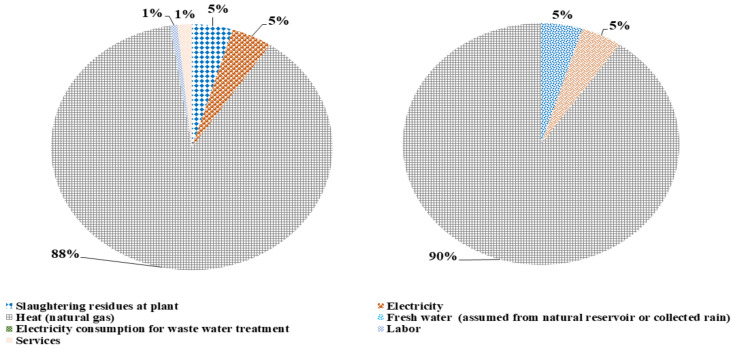
Emergy content, the contribution of different input flows for rendering I with and without L&S.

The total emergy content calculated for rendering I is 5.09E+18 seJ/yr and 4.97E+18 seJ/yr with L&S and without L&S, respectively. The emergy content contribution from each input to the total emergy content of the process has been shown in Figure 2. In the entire emergy content with L&S, the heat provision from natural gas has a maximum share contributing 87%, while services, electricity, and slaughtering residues have 2%, 5%, and 5% shares. Similarly, for the total emergy content, 90% share is contributed by heat from natural gas, the other 5% from electricity input, and 4% from slaughtering residues at the plant. This means that the raw material input has almost no relative contribution to the overall emergy content of the process. The utilities provide most of the emergy content to transform the raw material (waste residue) into a usable product.

### 3.3. Rendering II

The processing time of 1.60E+04 h/yr is required to process 2.36E+05 t/yr slaughtering residue following the same assumption described in rendering I. It is equivalent to a 9.97 person/yr labour force. Therefore, the sum of transportation service share and utility cost constitutes the service input for rendering I, 1.21E+07 €/yr. The inventory input and emergy accounting data are shown in Table 4.

**Figure 3:**
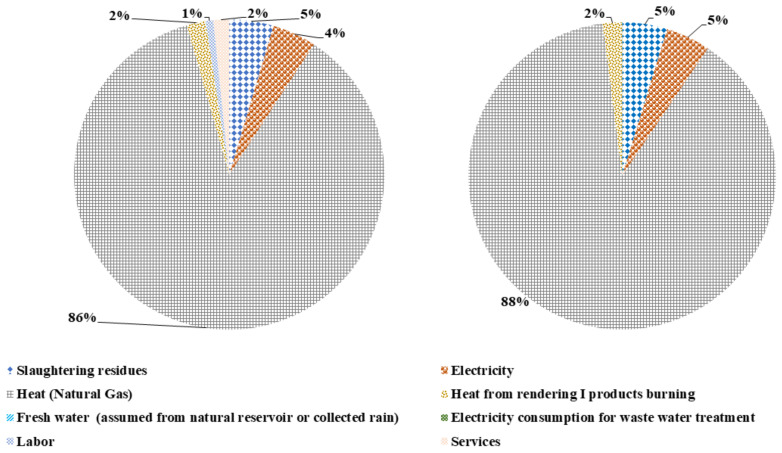
Emergy content share contribution of different input flows for rendering II with and without L&S.

The total emergy content for rendering II with L&S is 2.50E+20 seJ/yr, while without L&S it is 2.44E+20 seJ/yr. The emergy content contribution of different input flows for rendering II has been shown in Figure 3. In the total emergy content with L&S, heat provision by natural gas and rendering I have a cumulative share of 88%. The rest 12% is contributed by services (2%), electricity (5%), slaughtering residues (4%), and labour (1%), respectively. The total emergy content without L&S is also contributed mainly by heat provision from natural gas and rendering I. It contributes up to 90%, while the rest, 10%, is equally shared between electricity and slaughtering residues. It also shows a similar trend, as shown in Figure 3, for transforming waste raw material input into useable products tallow and MBM. The emergy content of the process is allocated to the products following the mass allocation method.

### 3.4. Biodiesel Production

The labour input for the biodiesel production facility is calculated by assuming that it has a capacity of 100,000 t/yr and it is operating 250 days/yr. Producing 3.31 t of biodiesel requires 1.95E+3 h/yr, equivalent to 1.22 person/yr labour force. The cost of raw material, chemicals, and utilities constitutes services equal to 7.03E+06 €/yr. The inventory data obtained from a project partner and emergy accounting calculations for the biodiesel production process with and without L&S has been presented in Table 5.

**Figure 4:**
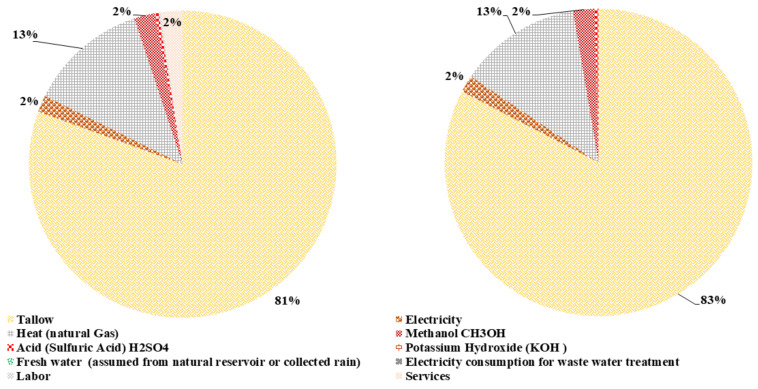
Contribution of different input flows for the emergy content of biodiesel production with and without L&S.

The graphical representation of the biodiesel production emergy content relevant to the material and service inputs is shown in Figure 4. The emergy content contributed by the tallow input is 81% and 83% of emergy content of biodiesel production with and without L&S. The provision of energy in the form of heat from natural gas has 13% and the electricity provision and methanol consumption has 2% of each emergy content contribution for both scenarios. The L&S contribute 2% emergy content in the total emergy value of biodiesel production with labour and services.

### 3.5. Hydrolysis

For a 10,000 t/yr PHA production, 150 batch/yr are required, requiring 1.2E+02 h/yr working time. It is equivalent to a 7.5E-01 person/yr labour force. Therefore, the sum of the cost for chemicals and utilities is equal to the services for this process, i.e., 4.22E+05 €/yr. The inventory input for hydrolysis and emergy content evaluation is shown in Table 6.

**Figure 5:**
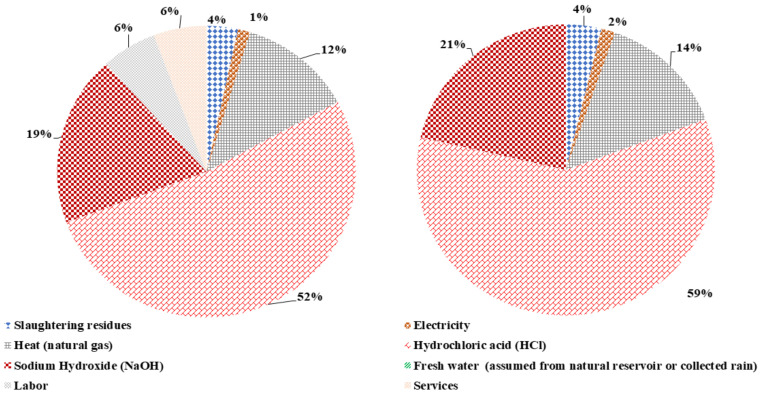
Contribution of different input flows for the emergy content of hydrolysis with and without L&S.

The total emergy content of hydrolysis for both scenarios, with and without L&S, is 2.49E+18 seJ/yr and 2.20E+18 seJ/yr, respectively. The emergy content contribution shown in Figure 5 reveals that the mineral acid and base are the key contributors in both scenarios.

Correspondingly, the mineral acid and base contribute 71% and 80% for the emergy content with L&S and without L&S. The heat provision input with 12% and 14% shares is the next prominent contributor in both scenarios. Finally, slaughtering waste has a small contribution of 3% and 4%, while the electricity input has an equal share of 2% in both systems. Thus, L&S have a considerable cumulative percentage of 12% in the total emergy content for hydrolysis with L&S.

### 3.6. Fermentation Process

The inventory data for the fermentation process and emergy evaluation for the fermentation process are given in Table 7. The published literature reveals that the fermentation batch and the handling of the downstream processing of the fermentation broth requires about 55 h/batch. There would be 150 batch/yr for 10,000 t/yr PHA production [38]. It results in 8.25E+03 h/yr, which is equivalent to 5.16 person/yr labour force. The sum of the cost for chemicals and utilities for the fermentation process is 6.31E+08 €/yr, equal to services input.

**Figure 6:**
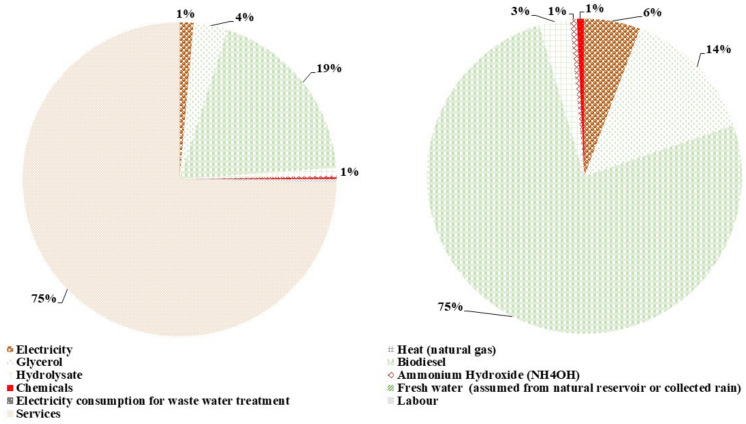
Contribution of input flows to the emergy content of the fermentation process with and without L&S.

The emergy content for a 10,000 t/yr PHA production with L&S is 2.88E+20 seJ/yr, while without L&S it is 7.34E+19 seJ/yr. The distribution of the total emergy content for the fermentation process with and without L&S into the emergy content of input shares is shown in Figure 6. The raw material’s (biodiesel and glycerol) emergy content cumulative contribution to the with L&S scenario is 22%, while in the without L&S scenario it is the primary input provider, contributing almost 85% of the content. For the total emergy content with the L&S scenario, services are the leading donor, having 74% of the overall emergy content, while electricity and hydrolysate contribute about 3%. Similarly, the emergy content contribution share for hydrolysate is 3%, for electricity its 6%, and for heat it is 4%. At the same time, ammonium hydroxide, chemicals, and freshwater have a cumulative 2% share in the total emergy content of fermentation without L&S. This shows that the raw material input has a significantly high emergy content contribution to the fermentation process. In contrast, the services’ input dominates the overall emergy content input.

### 3.7. Emergy-Based Performance Indicators

The emergy-based performance indicators calculated for the PHA production process, including the transportation phase and sub-processes like rendering I, rendering II, biodiesel production, and fermentation, have been shown in Table 8 using the system boundary at the biosphere level. The based performance indicators considering the PHA production facility area as the system boundary are given in Appendix A Table A1.

The emergy intensities of animal residue transportation, rendering I, rendering II, biodiesel production, hydrolysate, and PHA are 7.32E+07 seJ/g_animal residues transportation_, 1.25E+05 seJ/J_Heat_, 2.64E+09 seJ/g _(tallow, MBM)_, 2.96E+09 seJ/g_(biodiesel, glycerol)_, 6.80E+08 seJ/g_(hydrolysate)_, and 2.88E+10 seJ/g_(PHA)_, respectively. The ratio between the emergy content of imported resources (F) and the total emergy (U), i.e., “Emergy Yield Ratio”, is 1.0 for all sub-processes. This value shows that almost all resources are imported or transported from outside of the system under examination. The ELR is a ratio between the sum of emergy content of imported input “F”, labour “L”, and services “S” and renewable input “R”). The values of ELR for the sub-processes are 78.12 for the transportation phase, 12.58 and 12.58 for rendering I and rendering II, 13.87 for biodiesel production, 52.89 for hydrolysis, and 17.43 for the fermentation process. The values for ESI (ratio between EYR and ELR) are 0.01 for transportation, 0.08 for both rendering I and rendering II, 0.07 for biodiesel production, 0.02 for hydrolysis, and 0.06 for PHA production. The renewable fractions for different sub-processes are 1.26% for the transportation phase, 7.39% for rendering I, 7.36% for rendering II, 6.73% for biodiesel production, and 1.86% for hydrolysis 5.43% for the fermentation process.

### 3.8. Effect of the Change of Energy Provision Resource

A study about electricity production from different resources by Brown and Ulgiati (2002) [47] reveals % renewables from these energy systems. These % renewable values are also integrated as renewable input to the system under this study. The reported % renewable content for different energy resources are wind 86.61%, geothermal 69.67%, hydro 68.84%, natural gas (methane) 7.83%, and coal 8.79%.

#### 3.8.1. Comparative Analysis of Biodiesel Production

**Figure 7:**
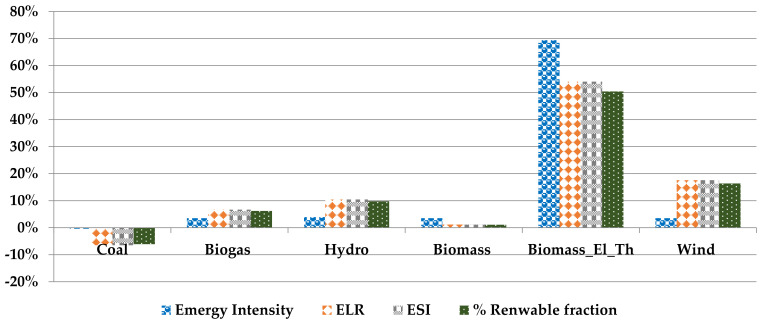
Comparative analysis of emergy flows and emergy-based indicators for the biodiesel production process.

The performance indicators calculated for the biodiesel production process fulfilling the energy demand from different energy resources are shown in Figure 7. The relevant values of this graph are given in Appendix A, Table A2.

The change of electricity provision resource shows an impact on different performance indicators. In the current study, the electricity provision form EU_27 mix is considered as the basic scenario, and the results calculated using other electricity provision systems are compared with it. The effect of the change of resource on the emergy intensity of biodiesel production (seJ/g) shows a negative impact on the electricity provision from coal while fulfilling the electricity demand using the electricity from biogas, hydro, biomass, and wind has a slightly positive effect, ranging between 3% to 4%. The provision of electricity and heat demand by biomass burning shows about a 69% improvement compared to the basic scenario of energy provision from the EU electricity mix and the natural gas for electricity and heat, respectively. The effect of changing energy resources on biodiesel transformity (seJ/J) compared to diesel transformity 1.81E+5 [45] has a positive impact with a 55% to 57% decrease in the transformity value for the electricity supply from the EU27 mix, coal, hydro power, wind, and biomass. In comparison, the electricity and heat provision from biomass has a maximum 87% decrease in the transformity value. The ELR value also shows a positive impact of −6% to 17% by replacing the electricity provision from more renewable resources. The change of electricity and heat provision source with biomass shows an improvement of the process of around 54%. Similarly, the ESI and % renewable fractions show an analogous effect, as both are dependent on ELR values.

#### 3.8.2. Comparative Analysis of PHA Production

**Figure 8:**
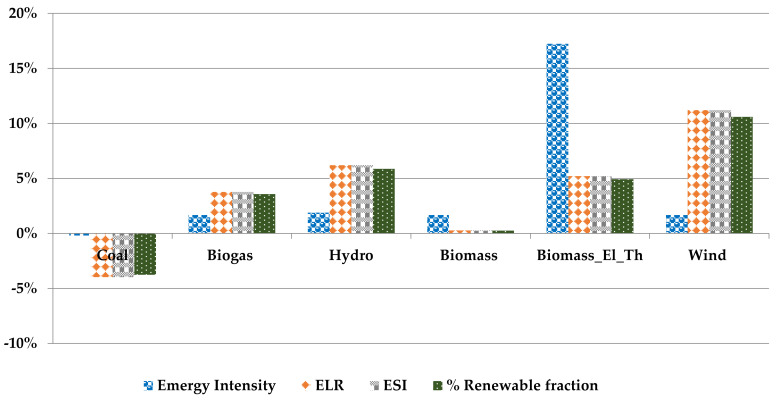
Comparative analysis of emergy flows and emergy-based indicators for the PHA production process.

The performance indicators calculated for the PHA production process providing the required energy demand from different energy resources have been shown in Figure 8. The corresponding computational values are given in Appendix A, Table A3.

The replacement of electricity provision with electricity from renewable resources shows a positive impact. The emergy intensity for PHA production (seJ/g) shows a minor improvement ranging from 1.5% to 2% by changing only the electricity provision resources. This impact reaches up to 17% when electricity and heat provision resources are replaced with biomass resources. The comparison of the emergy intensity for PHA production utilising the electricity provision from the EU27 mix, coal, hydropower, wind power, and biomass is about 5% to 7% lower than the emergy intensity of fossil-fuel-derived polyethylene high density (PE-HD) [53]. This decrease in the emergy intensity value is 21% lower when biomass is utilised to fulfil the PHA production process’s electric and heat provision. The exchange of electricity resources with electricity from coal negatively impacts the overall system, making it even less sustainable. The provision of electricity from wind farms represents the best available option, having the most significant improvements in ELR 11% and ESI 11%, and % a renewable fraction of 10.40%.

The ELR must be calculated regarding the local environment (R only calculated within the hectare of land occupied by the plant) compared to the regional scale (where the inputs come from, with imported flows divided into renewable non-renewable fractions). The ELR calculated in a second way compares all the renewable input flows at the larger scale (as the renewable fractions of all flows) with the non-renewable flows at the same scale. In the case of a system expansion from the local to the regional scale, the concept of imported flows F becomes irrelevant. The non-renewable fraction of F overlaps to non-renewable N. The calculated ELR has a meaning at the larger scale. The values of ELR calculated at the local scale become huge and indicate that the process has too much loading on the local resources. This shows that the process is not sustainable and becomes sustainable only if buffered by the environmental forces available at a large scale (therefore, power plants and industries need a forest, a protected area, around them. The ELR is linked to the %REN, which is higher in the large-scale perspective.

These choices do not affect the EYR values because F at the numerator and denominator has the same value, whether F is split into renewable or non-renewable fractions. EYR is an indicator that informs about the extent to which the process is exploiting the local resources (N and R). The value of the EYR indicator rises in case the exploitation of local resources increases. Otherwise, its value is 1, as in the current case study, and indicates a process that converts the imported resources into other resources for export. Similarly, ESI is affected because of its definition as a ratio of EYR and ELR.

#### 3.8.3. Evaluation of Waste

The emergy evaluation utilises local, regional, national, and biospheric boundary conditions that define the available renewable inputs to the system under consideration. Emergy dealing from a biosphere perspective has different meanings for anthropogenic waste terminology. It argues that no flow is a waste because every flow or residue from a process becomes an input to another process. In principle, any flow coming out as residue from a process is either a nutrient or a toxin, impacting the surrounding environment and leading to system evolution by favouring some processes and negatively affecting others. Similarly, Brown and Ulgiati (2010) [27] argued that the “*effect can be both positive and negative: Transformity does not suggest the outcome that might result from the interaction of a stressor with in an ecosystem, only that with high transformity the effect is greater. Where empower density of a stressor is significantly higher than the average empower density of the ecosystem it is released into, one can expect significant changes in ecosystem function*”. Similarly, the transformity of a product or service only explains the efficiencies of the current practices and compares optimisation potentials.

## 4. Conclusions

EMA evaluation results reveal that the main driving factors for hydrolysis are acid and base inputs. At the same time, heat provision also significantly affects the total emergy content of the process. Similarly, labour and services also have minor contributions to the entire emergy content of the sub-processes. The main contributing factor for rendering operations is heat provision, while residue transportation and electricity are minor contributors to the total emergy content of the process. For biodiesel production, the main contributor of emergy content is the tallow content, while heat provision is the minor contributor to the overall emergy content of the process. Tallow, a product of a highly energy-intensive rendering process and one of the main contributors of biodiesel production, indicates the possible potential of ecological optimisation by utilising energy from different resources. Similarly, the emergy accounting evaluation for the fermentation process shows that the main contributor to the total emergy content of this process is services, while biodiesel and glycerol also have significant contributions. EMA has the potential to rate energy technology. In this study, the exchange of energy from business as usual (EU mix grid electricity and natural gas for heating) to biomass for both heat and electricity represents the most environment-friendly option. The sustainability of biomass consumption as an energy source is in line with completing the short-term carbon cycle, including the fixation of CO_2_ by photosynthesis and releasing the same amount of CO_2_ in the combustion process. Similarly, in emergy accounting, wood or biomass are also accounted as renewable sources, which indicates that their replacement time is as fast as their use rate. The EMA, with real pluses and minuses, is a crucial methodology to attain global sustainability. There are a lot of methods available to assess the sustainable development of the process or products. It is up to the decision-makers (engineers in technology development especially) to look at their system boundary and select the suitable measurement methodology according to their needs. On the other hand, there are numerous uncertainties associated with renewable resource-based energy technologies, such as the location of the plant and the availability of renewable resources as a raw material for the plant and the energy production system. From the perspective of a chemical engineer, heat and energy integration in the design of new and existing plants may be different and more complex than state-of-the-art fossil-based energy systems.

## Figures and Tables

**Table 1:** Transformities and other Emergy intensities of material and energy flows.

Item	Value	Unit	Variations	References
	All flows are evaluated on a yearly basis. The numbers in the first column refer to the calculation procedures. UEV values refer to the 12E+24 baseline [31].			
**Renewable input (locally available)**				
Sun	1.00E+00	seJ/J	0%	[a]
Wind (kinetic wnergy of wind used at the surface)	2.51E+03	seJ/J	0%	[b]
Rainfall (chemical potential)	3.05E+04	seJ/J	0%	[b]
**Imported Input**				
Diesel for transport	1.81E+05	seJ/J	0%	[c]
Electricity	1.20E+05	seJ/J	0%	[d]
Heat (natural gas)	2.76E+05	seJ/J	0%	[c]
Heat (rendering I products combustion)	1.21E+05	seJ/J	0%	[e]
Biodiesel	2.86E+09	sej/g	0%	[e]
Glycerol	2.86E+09	sej/g	0%	[e]
Ammonium hydroxide (NH_4_OH)	6.38E+08	sej/g	0%	[f]
Chemicals	6.38E+08	sej/g	0%	[f]
Hydrochloric acid (HCl)	6.38E+08	sej/g	0%	[f]
Sodium hydroxide (NaOH)	6.38E+08	seJ/g	0%	[f]
Methanol CH_3_OH	6.38E+08	sej/g	0%	[f]
Acid (sulfuric acid) H_2_SO_4_	8.86E+08	seJ/g	0%	[g]
Potassium hydroxide (KOH)	6.38E+08	sej/g	0%	[f]
Hydrolysate	6.74E+08	sej/g	0%	[e]
Fresh water (assumed from natural reservoir or collected rain)	3.05E+04	seJ/J	0%	[b]
Wastewater treatment electricity consumption	1.49E+05	sej/g	0%	[d]
Emergy to money ratio for Austria, 2012	3.38E+11	seJ/€	0%	[h]
Labour	2.00E+17	seJ/capita	0%	[h]
Services	3.38E+11	seJ/€	0%	[h]

References for transformities: [a] By definition; [b] After Odum et al., 2000 [44]; [c] Brown et al., 2011. [45]; [d] own calculation after Brown & Ulgiati, 2001, 2002, 2004 [46,47,48] and Buonocore et al., 2012 [49]; [e] own calculation in this study; [f] After Odum et al., 2000 [50]; [g] Fahd and Fiorentino 2012 [51]; [h] our calculation after NEAD, 2014 [52].

**Table 2:** Emergy table of transportation phase.

Items	Units	Raw Amounts	Transformity (seJ/Unit)	Ref.	Emergy (seJ/yr)
Slaughtering residues	g/yr	2.43E+11	0.00E+00	[i]	0
Diesel for transport	J/yr	6.56E+13	1.81E+05	[c]	1.19E+19
Labour	working years	2.66E+01	2.00E+17	[h]	5.32E+18
Services	€/yr	1.74E+06	3.38E+11	[h]	5.89E+17
TOTAL EMERGY with L&S					1.78E+19
TOTAL EMERGY without L&S					1.19E+19

**Table 3:** Emergy table for rendering I.

Items	Units	Raw Amounts	Transformity (seJ/Unit)	Ref.	Emergy (seJ/yr)
Slaughtering residues at plant	g/yr	4.87E+09	4.89E+07	[f]	2.38E+17
Electricity EU_27 mix	J/yr	1.07E+12	2.58E+05	[d]	2.35E+17
Heat_natural gas	J/yr	1.61E+13	2.76E+05	[c]	4.45E+18
Fresh water (assumed from natural reservoir or collected rain)	J/yr	6.25E+09	3.05E+04	[b]	1.91E+14
Electricity consumption for waste water treatment	J/yr	2.09E+09	2.58E+05	[d]	5.10E+14
Labour	working years	2.05E-01	2.00E+17	[h]	4.11E+16
Services	€/yr	2.44E+05	3.38E+11	[h]	8.25E+16
TOTAL EMERGY with L&S					5.09E+18
TOTAL EMERGY without L&S					4.97E+18

**Table 4:** Emergy table of rendering II.

Items	Units	Raw Amounts	Transformity (seJ/Unit)	Ref.	Emergy (seJ/yr)
Slaughtering residues	g/yr	2.36E+11	4.89E+07	[f]	1.16E+19
Electricity	J/yr	5.19E+13	2.58E+05	[d]	1.14E+19
Heat (natural gas)	J/yr	7.83E+14	2.76E+05	[c]	2.16E+20
Heat from rendering I products burning	J/yr	4.07E+13	1.22E+05	[f]	4.93E+18
Fresh water (assumed from natural reservoir or collected rain)	J/yr	3.04E+11	3.05E+04	[b]	9.26E+15
Electricity consumption for waste water treatment	J/yr	4.76E+05	2.58E+05	[d]	1.05E+11
Labour	working years	9.97E+00	2.00E+17	[h]	1.99E+18
Services	€/yr	1.21E+07	3.38E+11	[h]	4.09E+18
TOTAL EMERGY with L&S					2.50E+20
TOTAL EMERGY without L&S					2.44E+20

**Table 5:** Emergy table of biodiesel production.

Items	Units	Raw Amounts	Transformity (seJ/Unit)	Ref.	Emergy (seJ/yr)
Tallow	g/yr	3.31E+10	2.57E+09	[e]	8.44E+19
Electricity	J/yr	8.46E+12	2.58E+05	[d]	1.86E+18
Heat (natural gas)	J/yr	4.77E+13	2.76E+05	[c]	1.32E+19
Methanol CH_3_OH	g/yr	3.61E+09	6.38E+08	[f]	2.30E+18
Acid (sulfuric acid) H_2_SO_4_	g/yr	4.63E+08	8.86E+08	[g]	4.10E+17
Potassium hydroxide (KOH)	g/yr	5.96E+05	6.38E+08	[f]	3.80E+14
Fresh water (assumed from natural reservoir or collected rain)	J/yr	1.63E+10	3.05E+04	[b]	4.99E+14
Electricity consumption for waste water treatment	J/yr	2.64E+09	2.58E+05	[d]	5.83E+14
Labour	working years	1.22E+00	2.00E+17	[h]	2.43E+17
Services	€/yr	7.03E+06	3.38E+11	[h]	2.38E+18
TOTAL EMERGY with L&S					1.05E+20
TOTAL EMERGY without L&S					1.02E+20

**Table 6:** Emergy table of hydrolysis.

Items	Units	Raw Amounts	Transformity (seJ/Unit)	Ref.	Emergy (seJ/yr)
Slaughtering residues	g/yr	1.73E+09	4.89E+07	[f]	8.44E+16
Electricity	J/yr	1.50E+11	2.58E+05	[d]	3.30E+16
Heat (natural gas)	J/yr	1.11E+12	2.76E+05	[c]	3.07E+17
Hydrochloric acid (HCl)	g/yr	2.04E+09	6.38E+08	[e]	1.30E+18
Sodium hydroxide (NaOH)	g/yr	7.36E+08	6.38E+08	[e]	4.70E+17
Fresh water (assumed from natural reservoir or collected rain)	J/yr	3.42E+09	3.05E+04	[b]	1.04E+14
Labour	working years	7.50E-01	2.00E+17	[h]	1.50E+17
Services	€/yr	4.22E+05	3.38E+11	[h]	1.43E+17
TOTAL EMERGY with L&S					2.49E+18
TOTAL EMERGY without L&S					2.20E+18

**Table 7:** Emergy table of fermentation (PHA production) process.

Items	Units	Raw Amounts	Transformity (seJ/Unit)	Ref.	Emergy (seJ/yr)
Electricity	g/yr	1.81E+13	2.20E+05	[d]	3.98E+18
Heat (natural gas)	J/yr	1.05E+13	2.76E+05	[c]	2.91E+18
Glycerol	J/yr	3.31E+09	2.93E+09	[f]	9.70E+18
Biodiesel	g/yr	1.78E+10	2.93E+09	[f]	5.23E+19
Hydrolysate	g/yr	3.67E+09	5.98E+08	[f]	2.19E+18
Ammonium hydroxide (NH_4_OH)	J/yr	7.67E+08	6.38E+08	[e]	4.89E+17
Chemicals	working years	7.82E+08	6.38E+08	[e]	4.99E+17
Fresh water (assumed from natural reservoir or collected rain)	€/yr	4.17E+11	3.05E+04	[b]	1.27E+16
Electricity consumption for waste water treatment	g/yr	6.74E+10	2.20E+05	[d]	1.48E+16
Labour	J/yr	5.16E+00	2.00E+17	[h]	1.03E+18
Services	€/yr	6.32E+08	3.38E+11	[h]	2.13E+20
TOTAL EMERGY with L&S					2.88E+20
TOTAL EMERGY without L&S					7.34E+19

**Table 8:** Emergy-based indicators calculated for the biobased PHA production overall process.

Emergy Accounting	Value	Unit
**Transportation phase**		
Emergy from local renewable resources, R	2.96E+17	seJ/yr
Emergy from imported resources, F	1.19E+19	seJ/yr
Total emergy, U = R + F + L + S	1.78E+19	seJ/yr
Emergy intensity	7.32E+07	seJ/g_animal residues transportation_
Environmental yield ratio, EYR = U/(F + L + S)	1.00	
Environmental Loading Ratio, (ELR) = (F + L + S)/R	78.12	
Emergy Sustainability Index, EYR/ELR	0.01	
Renewable fraction, REN% = 1/(1 + ELR) or =R/U × 100	1.26%	
**Rendering I**		
Emergy from local renewable resources, R	4.05E+17	seJ/yr
Emergy from imported resources, F	4.97E+18	seJ/yr
Total emergy, U = R + F + L + S	5.09E+18	seJ/yr
Transformity of heat	1.25E+05	seJ/J_Heat_
Environmental yield ratio, EYR = U/(F + L + S)	1.00	
Environmental Loading Ratio, (ELR) = (F + L + S)/R	12.54	
Emergy Sustainability Index, EYR/ELR	0.08	
Renewable fraction, REN% = 1/(1 + ELR) or =R/U × 100	7.39%	
**Rendering II**		
Emergy from local renewable resources, R	2.00E+19	seJ/yr
Emergy from imported resources, F	2.46E+20	seJ/yr
Total emergy, U = R + F + L + S	2.52E+20	seJ/yr
Emergy intensity	2.64E+09	seJ/g_(tallow, MBM)_
Environmental yield ratio, EYR = U/(F + L + S)	1.00	
Environmental Loading Ratio, (ELR) = (F + L + S)/R	12.58	
Emergy Sustainability Index, EYR/ELR	0.08	
Renewable fraction, REN% = 1/(1 + ELR) or =R/U × 100	7.36%	
**Biodiesel production**		
Emergy from local renewable resources, R	7.62E+18	seJ/yr
Emergy from imported resources, F	1.03E+20	seJ/yr
Total emergy, U = R + F + L + S	1.058E+20	seJ/yr
Emergy intensity of biodiesel	2.96E+09	seJ/g_(biodiesel, glycerol)_
Environmental yield ratio, EYR = U/(F + L + S)	1.00	
Environmental Loading Ratio, (ELR) = (F + L + S)/R	13.87	
Emergy Sustainability Index, EYR/ELR	0.07	
Renewable fraction, REN% = 1/(1 + ELR) or =R/U × 100	6.73%	
**Hydrolysis**		
Emergy from local renewable resources, R	4.69E+16	seJ/yr
Emergy from imported resources, F	7.05E+19	seJ/yr
Total emergy, U = R + F + L + S	2.49E+18	seJ/yr
Emergy intensity of hydrolysate	6.80E+08	seJ/g_(hydrolysate)_
Environmental yield ratio, EYR = U/(F + L + S)	1.00	
Environmental Loading Ratio, (ELR) = (F + L + S)/R	52.89	
Emergy Sustainability Index, EYR/ELR	0.02	
Renewable fraction, REN% = 1/(1 + ELR) or =R/U × 100	1.86%	
**Fermentation (PHA production) process**		
Emergy from local renewable resources, R	1.59E+19	seJ/yr
Emergy from imported resources, F	7.34E+19	seJ/yr
Total emergy, U = R + F + L + S	2.88E+20	seJ/yr
Emergy intensity of PHA	2.88E+10	seJ/g_PHA_
Environmental yield ratio, EYR = U/(F + L + S)	1.00	
Environmental Loading Ratio, (ELR) = (F + L + S)/R	17.43	
Emergy Sustainability Index, EYR/ELR	0.06	
Renewable fraction, REN% = 1/(1+ELR) or =R/U × 100	5.43%	

## Data Availability

Not applicable.

## Appendix A

**Table A1:** Emergy-based indicators calculated for the biobased PHA production using facility area as the system boundary.

Emergy Accounting	Value	Unit
**Transportation Phase**		
Emergy from local renewable resources, R	4.34E+14	seJ/yr
Emergy from imported resources, F	1.19E+19	seJ/yr
Total emergy, U = R + F + L + S	1.78E+19	seJ/yr
Emergy intensity	7.32E+07	seJ/g_animal residues_
Environmental yield ratio, EYR = U/(F + L + S)	1.00	
Environmental Loading Ratio, (ELR) = (F + L + S)/R	40956.33	
Emergy Sustainability Index, EYR/ELR	0.00002	
Renewable fraction, REN% = 1/(1 + ELR) or =R/U × 100	0.0024%	
**Rendering I**		
Emergy from local renewable resources, R	4.34E+14	seJ/yr
Emergy from imported resources, F	4.82E+18	seJ/yr
Total emergy, U = R + F + L + S	4.94E+18	seJ/yr
Transformity of heat	1.21E+05	seJ/J_Heat_
Environmental yield ratio, EYR = U/(F + L + S)	1.00	
Environmental Loading Ratio, (ELR) = (F + L + S)/R	11382.85	
Emergy Sustainability Index, EYR/ELR	0.0001	
Renewable fraction, REN% = 1/(1 + ELR) or =R/U × 100	0.0088%	
**Rendering II**		
Emergy from local renewable resources, R	4.34E+14	seJ/yr
Emergy from imported resources, F	2.39E+20	seJ/yr
Total emergy, U = R + F + L + S	2.45E+20	seJ/yr
Emergy intensity	2.56E+09	seJ/g_(tallow, MBM)_
Environmental yield ratio, EYR = U/(F + L + S)	1.00	
Environmental Loading Ratio, (ELR) = (F + L + S)/R	564420.33	
Emergy Sustainability Index, EYR/ELR	0.000002	
Renewable fraction, REN% = 1/(1 + ELR) or =R/U × 100	0.0002%	
**Biodiesel production**		
Emergy from local renewable resources, R	4.34E+14	seJ/yr
Emergy from imported resources, F	9.95E+19	seJ/yr
Total emergy, U = R + F + L + S	1.021E+20	seJ/yr
Emergy intensity	2.86E+09	seJ/g_(biodiesel, glycerol)_
Environmental yield ratio, EYR = U/(F + L + S)	1.00	
Environmental Loading Ratio, (ELR) = (F + L + S)/R	235228.24	
Emergy Sustainability Index, EYR/ELR	0.0000043	
Renewable fraction, REN% = 1/(1 + ELR) or =R/U × 100	0.00043%	
**Hydrolysis**		
Emergy from local renewable resources, R	4.34E+14	seJ/yr
Emergy from imported resources, F	2.18E+18	seJ/yr
Total emergy, U = R + F + L + S	2.47E+18	seJ/yr
Emergy intensity of hydrolysate	6.74E+08	seJ/g_(hydrolysate)_
Environmental yield ratio, EYR = U/(F + L + S)	1.00	
Environmental Loading Ratio, (ELR) = (F + L + S)/R	5695.91	
Emergy Sustainability Index, EYR/ELR	0.00018	
Renewable fraction, REN% = 1/(1+ELR) or =R/U × 100	0.018%	
**Fermentation (PHA production) process**		
Emergy from local renewable resources, R	4.34E+14	seJ/yr
Emergy from imported resources, F	6.58E+19	seJ/yr
Total emergy, U = R + F + L + S	2.74E+20	seJ/yr
Emergy intensity of PHA	2.74E+10	seJ/g_PHA_
Environmental yield ratio, EYR = U/(F + L + S)	1.00	
Environmental Loading Ratio, (ELR) = (F + L + S)/R	630188.11	
Emergy Sustainability Index, EYR/ELR	0.0000016	
Renewable fraction, REN% = 1/(1 + ELR) or =R/U × 100	0.00016%	

**Table A2:** Comparative analytic values of emergy flows and emergy-based indicators for the biodiesel production process using alternate energy resources.

Indicators	EU_27	Coal	Biogas	Hydro	Biomass	Biomass_El_Th	Wind
Emergy from local renewable resources, R	7.62E+18	7.18E+18	7.88E+18	8.17E+18	7.44E+18	5.06E+18	8.92E+18
Emergy from imported resources, F	1.03E+20	1.04E+20	9.95E+19	9.90E+19	9.95E+19	2.98E+19	9.95E+19
Total emergy, U = R + F + L + S	1.06E+20	1.06E+20	1.02E+20	1.02E+20	1.02E+20	3.24E+19	1.02E+20
Emergy intensity	2.96E+09	2.97E+09	2.86E+09	2.84E+09	2.86E+09	9.06E+08	2.86E+09
Environmental yield ratio, EYR = U/(F + L + S)	1.00	1.00	1.00	1.00	1.00	1.00	1.00
Environmental Loading Ratio, (ELR) = (F + L + S)/R	13.87	14.77	12.94	12.42	13.71	6.37	11.43
Emergy Sustainability Index, EYR/ELR	0.072	0.068	0.077	0.081	0.073	0.157	0.087
Renewable fraction, REN% = 1/(1 + ELR) or =R/U × 100	6.73%	6.34%	7.17%	7.45%	6.80%	13.56%	8.05%
Percentage deviations:							
Emergy intensity		−0.39%	3.50%	3.95%	3.50%	69.40%	3.50%
ELR		−6.51%	6.68%	10.45%	1.17%	54.03%	17.58%
ESI = EYR/ELR		−6.51%	6.68%	10.45%	1.17%	54.03%	17.58%
% Renewable fraction		−6.08%	6.23%	9.75%	1.09%	50.40%	16.40%

Reference: Brown and Ulgiati 2002.

**Table A3:** Comparative analytic values of emergy flows and emergy-based indicators for the PHA production process using alternate energy resources.

Indicators	EU_27	Coal	Biogas	Hydro	Biomass	Biomass_El_Th	Wind
Emergy from local renewable resources, R	1.59E+19	1.53E+19	1.62E+19	1.66E+19	1.57E+19	1.38E+19	1.76E+19
Emergy from imported resources, F	7.34E+19	7.39E+19	6.87E+19	6.81E+19	6.87E+19	2.49E+19	6.87E+19
Total emergy, U = R + F + L + S	2.88E+20	2.88E+20	2.83E+20	2.83E+20	2.83E+20	2.39E+20	2.83E+20
Emergy intensity	2.88E+10	2.88E+10	2.83E+10	2.83E+10	2.83E+10	2.39E+10	2.83E+10
Environmental yield ratio, EYR = U/(F + L + S)	1.00	1.00	1.00	1.00	1.00	1.00	1.00
Environmental Loading Ratio, (ELR) = (F + L + S)/R	17.43	18.11	16.78	16.37	17.38	16.55	15.51
Emergy Sustainability Index, EYR/ELR	0.057	0.055	0.060	0.061	0.058	0.060	0.064
Renewable fraction, REN% = 1/(1 + ELR) or =R/U × 100	5.43%	5.23%	5.62%	5.76%	5.44%	5.70%	6.06%
**Percentage deviation:**							
Emergy intensity		−0.18%	1.64%	1.85%	1.64%	16.84%	1.64%
ELR		−3.89%	3.70%	6.08%	0.27%	5.05%	11.00%
ESI = EYR/ELR		−3.89%	3.70%	6.08%	0.27%	5.05%	11.00%
% Renewable fraction		−3.68%	3.50%	5.75%	0.25%	4.78%	10.40%

Reference: Brown and Ulgiati 2002.

## Appendix B

### Appendix B.1. Emergy Nomenclature

(From: Odum, H. T., 1996. “Environmental Accounting”. J. Willey.)

**Symbol**	**Description**
	**Energy circuit:** A pathway whose flow is proportional to the quantity in the storage or source upstream.
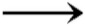
	**Source:** Outside source of energy-delivering forces according to a programme controlled from the outside: a forcing function.
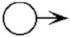
	**Tank:** A compartment of energy storage within the system storing a quantity as the balance of inflows and outflows. It is a state variable.
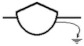
	**Heat sink:** Dispersion of potential energy into heat that accompanies all fundamental transformation processes and storages, loss of potential energy from further use by the system.
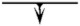
	**Interaction:** Interactive intersection of two pathways coupled to produce an outflow in proportion to a function of both control actions of one flow on another limiting factor action work gate.
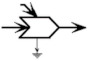
	**Consumer unit** that transforms energy quality, stores it, and feeds it back autocatalytically to improve the inflow.
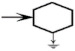
	**Switching action:** A symbol that indicates one or more switching actions
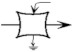
	**Producer unit** that collects and transforms low-quality energy under control interactions of high-quality flows
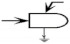
	**Self-limiting energy receiver:** A unit that has a self-limiting output when input drives are high because there is a limiting constant quality of material reacting on a circular pathway within.
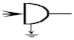
	**Box:** Miscellaneous symbol to use for whatever unit or function is labelled.
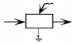
	**Constant-gain amplifier.** A unit that delivers an output in proportion to the input I but is changed by a constant factor as long as the energy source S is sufficient
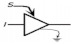
	**Transaction:** A unit that indicates a sale of goods or services (solid line) in exchange for the payment of money (dashed line). The price is shown as an external source.
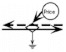

### Appendix B.2. Definitions of Terms

**Available energy**	Potential energy capable of doing work and being degraded in the process (units: kilocalories, Joules, etc.)
**Useful energy**	Available energy used to increase system production and efficiency. Power- Useful energy flow per unit time
**Emergy**	Available energy (exergy) of one kind previously required directly and indirectly to make a product or service (units: emjoules, emkilocalories, etc.)
**Empower**	Emergy flow per unit time (units: emjoules per unit time) Transformity-emergy per unit of available energy (units: emjoule per joule)
**Solar emergy**	Solar energy required directly and indirectly to make a product or service (units: solar emjoules)
**Solar empower**	Solar emergy flow per unit time (units: solar emjoules per unit time)
**Emergy intensity**	Emergy of one kind required to produce a product or service per unit of output of the product or service. There are two types of EIs: transformity and specific emergy
**Solar transformity:**	Solar emergy per unit of available energy (units: solar emjoules per Joule)
**Specific emergy (solar)**	Solar emergy per mass of a product (units: solar emjoules per gram)
**Emdollars, (Em$)**	Dollars of gross economic product due to an emergy contribution’s proportion of the national empower
(After Odum, 1996).	
